# Supplementary figures and images for: ggPlantmap: an open-source R package for the creation of informative and quantitative ggplot maps derived from plant images
Source: J Exp Bot. 2024 Feb 8;75(17):5366–76. doi: 10.1093/jxb/erae043 (PMC11389834; doi:10.1093/jxb/erae043)

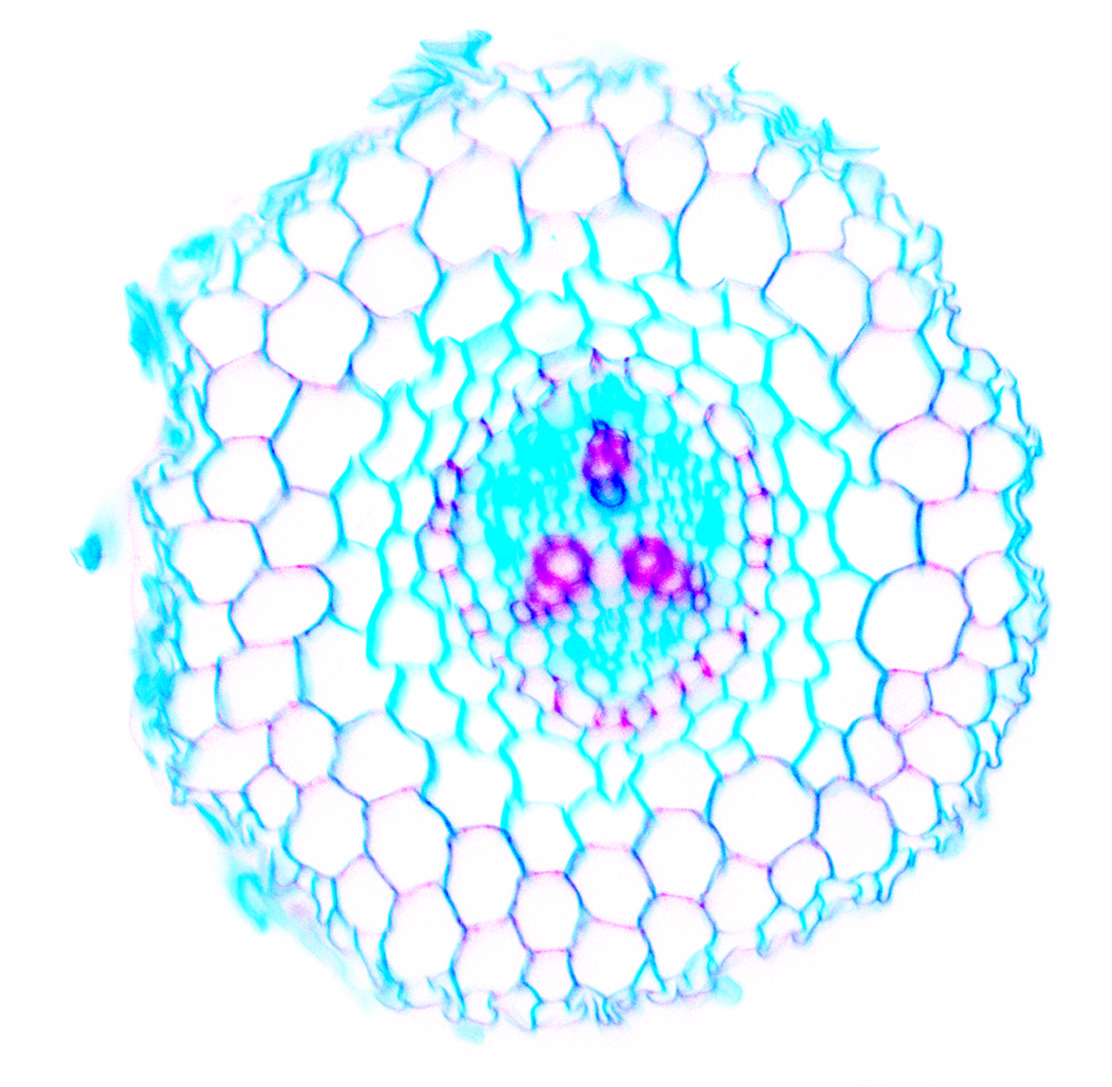

Supplement: erae043_suppl_Supplementary_Datasets_S1 [file erae043_suppl_supplementary_datasets_s1.zip › sample.jpg]
